# EasyMetagenome: A user‐friendly and flexible pipeline for shotgun metagenomic analysis in microbiome research

**DOI:** 10.1002/imt2.70001

**Published:** 2025-02-14

**Authors:** Defeng Bai, Tong Chen, Jiani Xun, Chuang Ma, Hao Luo, Haifei Yang, Chen Cao, Xiaofeng Cao, Jianzhou Cui, Yuan‐Ping Deng, Zhaochao Deng, Wenxin Dong, Wenxue Dong, Juan Du, Qunkai Fang, Wei Fang, Yue Fang, Fangtian Fu, Min Fu, Yi‐Tian Fu, He Gao, Jingping Ge, Qinglong Gong, Lunda Gu, Peng Guo, Yuhao Guo, Tang Hai, Hao Liu, Jieqiang He, Zi‐Yang He, Huiyu Hou, Can Huang, Shuai Ji, ChangHai Jiang, Gui‐Lai Jiang, Lingjuan Jiang, Ling N. Jin, Yuhe Kan, Da Kang, Jin Kou, Ka‐Lung Lam, Changchao Li, Chong Li, Fuyi Li, Liwei Li, Miao Li, Xin Li, Ye Li, Zheng‐Tao Li, Jing Liang, Yongxin Lin, Changzhen Liu, Danni Liu, Fengqin Liu, Jia Liu, Tianrui Liu, Tingting Liu, Xinyuan Liu, Yaqun Liu, Bangyan Liu, Minghao Liu, Wenbo Lou, Yaning Luan, Yuanyuan Luo, Hujie Lv, Tengfei Ma, Zongjiong Mai, Jiayuan Mo, Dongze Niu, Zhuo Pan, Heyuan Qi, Zhanyao Shi, Chunjiao Song, Fuxiang Sun, Yan Sun, Sihui Tian, Xiulin Wan, Guoliang Wang, Hongyang Wang, Hongyu Wang, Huanhuan Wang, Jing Wang, Jun Wang, Kang Wang, Leli Wang, Shao‐kun Wang, Xinlong Wang, Yao Wang, Zufei Xiao, Huichun Xing, Yifan Xu, Shu‐yan Yan, Li Yang, Song Yang, Yuanming Yang, Xiaofang Yao, Salsabeel Yousuf, Hao Yu, Yu Lei, Zhengrong Yuan, Meiyin Zeng, Chunfang Zhang, Chunge Zhang, Huimin Zhang, Jing Zhang, Na Zhang, Tianyuan Zhang, Yi‐Bo Zhang, Yupeng Zhang, Zheng Zhang, Mingda Zhou, Yuanping Zhou, Chengshuai Zhu, Lin Zhu, Yue Zhu, Zhihao Zhu, Hongqin Zou, Anna Zuo, Wenxuan Dong, Tao Wen, Shifu Chen, Guoliang Li, Yunyun Gao, Yong‐Xin Liu

**Affiliations:** ^1^ Genome Analysis Laboratory of the Ministry of Agriculture and Rural Affairs, Agricultural Genomics Institute at Shenzhen, Chinese Academy of Agricultural Sciences Shenzhen Guangdong China; ^2^ State Key Laboratory for Quality Ensurance and Sustainable Use of Dao‐di Herbs, National Resource Center for Chinese Materia Medica, China Academy of Chinese Medical Sciences Beijing China; ^3^ School of Horticulture Anhui Agricultural University Hefei China; ^4^ College of Life Sciences Qingdao Agricultural University Qingdao China; ^5^ Key Laboratory for Bio‐Electromagnetic Environment and Advanced Medical Theranostics, School of Biomedical Engineering and Informatics Nanjing Medical University Nanjing Jiangsu China; ^6^ Center for Water and Ecology, State Key Joint Laboratory of Environment Simulation and Pollution Control, School of Environment Tsinghua University Beijing China; ^7^ Immunology Translational Research Programme, Yong Loo Lin School of Medicine National University of Singapore Singapore Singapore; ^8^ Research Center for Parasites and Vectors, College of Veterinary Medicine Hunan Agricultural University Changsha Hunan China; ^9^ Institute of Marine Biology and Pharmacology, Ocean College Zhejiang University Zhoushan Zhejiang China; ^10^ Agro‐Environmental Protection Institute Ministry of Agriculture and Rural Affairs Tianjin China; ^11^ Key Laboratory for Molecular Genetic Mechanisms and Intervention Research on High Altitude Disease of Tibet Autonomous Region, School of Medicine Xizang Minzu University Xianyang China; ^12^ Karolinska Institutet, Department of Microbiology, Tumor and Cell Biology Stockholm Sweden; ^13^ College of Environment Zhejiang University of Technology Hangzhou China; ^14^ College of Environmental and Resource Sciences Zhejiang Agriculture and Forestry University Hangzhou China; ^15^ The College of Forestry Beijing Forestry University Beijing China; ^16^ Department of Bioinformatics, Hangzhou VicrobX Biotech Co., Ltd Hangzhou Zhejiang China; ^17^ Anhui Province Key Laboratory of Integrated Pest Management on Crops, College of Plant Protection Anhui Agricultural University Hefei China; ^18^ Xiangya School of Basic Medicine Central South University Changsha Hunan China; ^19^ Institute of Microbiology,Guangdong Academy of Sciences Guangzhou Guangdong China; ^20^ Engineering Research Center of Agricultural Microbiology Technology, Ministry of Education, School of Life Sciences Heilongjiang University Harbin China; ^21^ College of Animal Science and Technology Jilin Agricultural University Changchun Jilin China; ^22^ Sansure Biotech Incorporation Changsha Hunan China; ^23^ School of Food Science and Biology Hebei University of Science and Technology Shijiazhuang Hebei China; ^24^ School of Life Sciences Shanxi Datong University Datong China; ^25^ Department of Health & Environmental Sciences Xi'an Jiaotong‐Liverpool University Suzhou Jiangsu China; ^26^ College of Horticulture Northwest A&F University Yangling Shaanxi China; ^27^ School of Agriculture, Food and Ecosystem Sciences, Faculty of Science The University of Melbourne VIC Australia; ^28^ Graduate School of Frontier Sciences The University of Tokyo Kashiwa‐shi, Chiba Japan; ^29^ Institute of Biotechnology, Helsinki Institute of Life Science University of Helsinki Helsinki Finland; ^30^ Shanghai Maritime University Shanghai China; ^31^ Suzhou Medical College Soochow University Suzhou Jiangsu China; ^32^ Biomarker Discovery and Validation Facility, Institute of Clinical Medicine, Peking Union Medical College Hospital Beijing China; ^33^ Department of Civil and Environmental Engineering The Hong Kong Polytechnic University Hong Kong China; ^34^ College of Biology and Oceanography Weifang University Weifang Shandong China; ^35^ College of Environmental Science and Engineering Beijing University of Technology Beijing China; ^36^ College of Environmental and Municipal Engineering Lanzhou Jiaotong University Lanzhou China; ^37^ School of Life Sciences The Chinese University of Hong Kong Shatin, Hong Kong China; ^38^ Department of Renewable Resources University of Alberta Edmonton Alberta Canada; ^39^ School of Geographical Sciences Northeast Normal University Changchun Jilin China; ^40^ Department of Gastroenterology The Second Affiliated Hospital of Guangxi Medical University Nanning Guangxi China; ^41^ Synaura Biotechnology (Shanghai) Co., Ltd. Shanghai China; ^42^ School of Public Health University of Michigan Ann Arbor Michigan USA; ^43^ Institute of Soil Science, Chinese Academy of Sciences Nanjing Jiangsu China; ^44^ School of Art and Archaeology of Zhejiang University Zhejiang China; ^45^ College of Animal Science and Technology Guangxi University Nanning China; ^46^ Fujian Provincial Key Laboratory for Subtropical Resources and Environment Fujian Normal University Fuzhou China; ^47^ College of Energy and Environmental Engineering Hebei University of Engineering Handan Hebei China; ^48^ HaploX Biotechnology Shenzhen China; ^49^ College of Life Sciences Henan Agricultural University Zhengzhou China; ^50^ College of Life Science Nankai University Tianjin China; ^51^ Jiangxi Province Key Laboratory of Sustainable Utilization of Traditional Chinese Medicine Resources, Institute of Traditional Chinese Medicine Health Industry, China Academy of Chinese Medical Sciences Jiangxi China; ^52^ Beijing Key Laboratory of Emerging Infectious Diseases, Institute of Infectious Diseases, Beijing Ditan Hospital Capital Medical University Beijing China; ^53^ State Key Laboratory of Tea Plant Biology and Utilization Anhui Agricultural University Hefei Anhui China; ^54^ School of Life Sciences and Food Technology Hanshan Normal University Chaozhou China; ^55^ Tsinghua University Beijing China; ^56^ State Key Laboratory of Microbial Resources, Institute of Microbiology, Chinese Academy of Sciences Beijing China; ^57^ Department of Life Sciences, Imperial College of London London UK; ^58^ State Key Laboratory of Herbage Improvement and Grassland Agro‐Ecosystems, Centre for Grassland Microbiome, College of Pastoral Agriculture Science and Technology Lanzhou University Lanzhou Gansu China; ^59^ Department of Oncology The Fifth Affiliated Hospital of Sun Yat‐sen University Zhuhai Guangdong China; ^60^ National‐Local Joint Engineering Research Center of Biomass Refining and High‐Quality Utilization, Institute of Urban and Rural Mining Changzhou University Changzhou Jiangsu China; ^61^ Department of Pathology Affiliated Cancer Hospital of Zhengzhou University Zhengzhou China; ^62^ Institute of Microbiology, Chinese Academy of Sciences Beijing China; ^63^ College of Water Sciences Beijing Normal University Beijing China; ^64^ Deyang People's Hospital Deyang Sichuan China; ^65^ New Direction Biotechnology (Tianjin) Co., Ltd Tianjin China; ^66^ College of Energy and Environmental Engineering, Hebei Key Laboratory of Air Pollution Cause and Impact Hebei University of Engineering Handan China; ^67^ Institute of Botany, Chinese Academy of Sciences Beijing China; ^68^ Institute of Biotechnology, Beijing Academy of Agriculture and Forestry Sciences Beijing China; ^69^ National Resource Center for Chinese Materia Medica, China Academy of Chinese Medical Sciences Jiangsu China; ^70^ College of Animal Science Anhui Science and Technology University Chuzhou China; ^71^ State Key Laboratory for Biology of Plant Diseases and Insect Pests, Institute of Plant Protection, Chinese Academy of Agricultural Sciences Beijing China; ^72^ State Key Laboratory of Environmental Criteria and Risk Assessment, Chinese Research Academy of Environmental Sciences Beijing China; ^73^ China CDC Key Laboratory of Environment and Population Health, National Institute of Environmental Health, Chinese Center for Disease Control and Prevention Beijing China; ^74^ College of Animal Science and Technology Yangzhou University Yangzhou Jiangsu China; ^75^ Key Laboratory of Agro‐Ecological Processes in Subtropical Region, Institute of Subtropical Agriculture, Chinese Academy of Sciences Changsha China; ^76^ Institute of Ecological Conservation and Restoration, Chinese Academy of Forestry Beijing China; ^77^ State Key Laboratory for Ecological Security of Regions and Cities, Institute of Urban Environment, Chinese Academy of Sciences Xiamen China; ^78^ Center of Liver Diseases Division 3, Beijing Ditan Hospital Capital Medical University Beijing China; ^79^ State Key Laboratory for Biology of Plant Diseases and Insect Pests, Key Laboratory of Invasive Alien Species Control of Ministry of Agriculture and Rural Affairs, Institute of Plant Protection, Chinese Academy of Agricultural Sciences Beijing China; ^80^ Guangzhou University of Chinese Medicine Guangzhou China; ^81^ Key Laboratory of Livestock Biology Northwest A&F University Yangling Shaanxi China; ^82^ College of Biological Sciences and Technology Beijing Forestry University Beijing China; ^83^ CAS Key Laboratory of Pathogenic Microbiology and Immunology, Institute of Microbiology, Chinese Academy of Sciences Beijing China; ^84^ School of Food Science and Technology Shihezi University Shihezi Xinjiang China; ^85^ College of Biochemical Engineering Beijing Union University Beijing China; ^86^ College of Resources and Environmental Sciences Henan Agricultural University Zhengzhou China; ^87^ Tea Research Institute, Chinese Academy of Agricultural Sciences Hangzhou Zhejiang China; ^88^ College of Environmental Science and Engineering Tongji University Shanghai China; ^89^ Zhanjiang Key Laboratory of Human Microecology and Clinical Translation Research, the Marine Biomedical Research Institute, College of Basic Medicine Guangdong Medical University Zhanjiang Guangdong China; ^90^ State Key Laboratory of Urban Water Resource and Environment, School of Environment, Harbin Institute of Technology Harbin China; ^91^ School of Ecology, Environment and Resources Guangdong University of Technology Guangzhou Guangdong China; ^92^ Institute of Agricultural Resources and Regional Planning, Chinese Academy of Agricultural Sciences Beijing China; ^93^ School of Traditional Chinese Medicine Southern Medical University Guangzhou Guangdong China; ^94^ Department of Animal Sciences Purdue University West Lafayette Indiana USA; ^95^ College of Resource and Environmental Sciences Nanjing Agricultural University Nanjing Jiangsu China; ^96^ LifeX Institute, School of Medical Technology Gannan Medical University Ganzhou China; ^97^ Faculty of Data Science City University of Macau Macau China; ^98^ Jiangxi Provincial Key Laboratory of Conservation Biology, College of Forestry Jiangxi Agricultural University Nanchang Jiangxi China

**Keywords:** metagenome, microbiome, microbiota, pipeline, visualization

## Abstract

Shotgun metagenomics has become a pivotal technology in microbiome research, enabling in‐depth analysis of microbial communities at both the high‐resolution taxonomic and functional levels. This approach provides valuable insights of microbial diversity, interactions, and their roles in health and disease. However, the complexity of data processing and the need for reproducibility pose significant challenges to researchers. To address these challenges, we developed EasyMetagenome, a user‐friendly pipeline that supports multiple analysis methods, including quality control and host removal, read‐based, assembly‐based, and binning, along with advanced genome analysis. The pipeline also features customizable settings, comprehensive data visualizations, and detailed parameter explanations, ensuring its adaptability across a wide range of data scenarios. Looking forward, we aim to refine the pipeline by addressing host contamination issues, optimizing workflows for third‐generation sequencing data, and integrating emerging technologies like deep learning and network analysis, to further enhance microbiome insights and data accuracy. EasyMetageonome is freely available at https://github.com/YongxinLiu/EasyMetagenome.

## Highlights

- EasyMetagenome offers multiple installation options and detailed question and answer explanations.
- EasyMetagenome includes detailed parameter explanations to accommodate various data scenarios, ensuring flexibility and adaptability.
- EasyMetagenome supports comprehensive downstream analysis from raw data, including read‐based, assembly‐based, binning, and genome analysis, with the ability to generate publication‐ready visualizations.
- EasyMetagenome is developed as an open‐source project, encouraging collaboration from the community. It's available for access and contribution at https://github.com/YongxinLiu/EasyMetagenome.

## Graphical abstract

EasyMetagenome is a user‐friendly shotgun metagenomics pipeline designed for comprehensive microbiome analysis, supporting quality control, host removal, read‐based, assembly‐based, binning, genome and pan‐genome analysis. It offers customizable settings, data visualizations, and parameter explanations. The pipeline is freely available at https://github.com/YongxinLiu/EasyMetagenome.

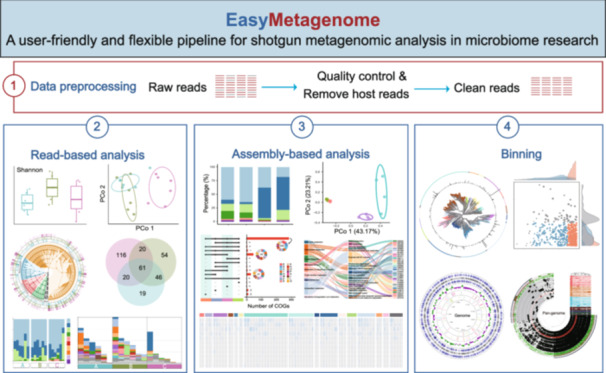

## INTRODUCTION

Microorganisms, including bacteria, archaea, fungi, viruses, and protists, are ubiquitous in natural environments [1, 2, 3]. The term “microbiome” broadly refers to the community of microorganisms and their genomes within a particular host or environment [4]. Microbiome research has significantly expanded our understanding across various fields, including agriculture, food science, biotechnology, bio‐economics, animal and plant nutrition and health, and particularly in human medicine [5, 6]. Since the release of the first shotgun metagenome from environmental microbiota samples [7], microbiome research has grown explosively in both data generation and methodological advancements.

Unlike amplicon sequencing, which typically targets specific genetic regions (such as 16S rRNA genes), shotgun metagenomics (also known as short‐read, second‐generation, next‐generation, or high‐throughput sequencing) captures all DNA in a sample. It can provide a relatively unbiased view of the complete taxonomic composition and functional potential of microbial communities [8, 9]. Additionally, it enables the recovery of metagenomic assembled genomes (MAGs), which offer insights into microbial diversity with high taxonomic resolution—down to the species level and, in some cases, even the strain level [10]. Therefore, shotgun metagenomic sequencing has become a transformative tool in microbiome research, greatly enhancing our understanding of microbial communities in humans and other environments [11, 12]. Although the development of third‐generation long‐read sequencing has helped address some limitations of short‐read sequencing, its higher error rate and lower throughput mean that most studies still rely on hybrid approaches (combining long‐read and short‐read data) for error correction [13, 14, 15, 16]. Thus, second‐generation metagenomic sequencing remains crucial in microbiome research.

After generating shotgun metagenomic data from a microbial community of interest, the typical analysis begins with quality control and removal of host DNA, followed by profiling taxonomic, functional and genomic features of the microbiome [8, 17], to gain insights into the underlying biology. Various tools, R packages, and pipelines have been developed to accomplish each stage of the analysis. For example, fastp [18] or Trimmomatic [19] are used for basic quality control, Bowtie2 [20] or KneadData (https://huttenhower.sph.harvard.edu/kneaddata) are employed for removing reads that map to the host reference database, Kraken2 [21] and Bracken [22] are used for taxonomic profiling, HUMAnN3 [23] are utilized for generating gene families and MetaCyc [24] pathways, MEGAHIT [25] or MetaSPAdes [26] serve as metagenome assemblers, and MetaWRAP [27] is used for extracting draft metagenome‐assembled genomes (MAGs) from metagenomes, among others. Also, plenty of R packages for exploring microbial community diversity, structure, and potential functions [10, 28]. Each of these tools or R packages has multiple parameters or reference databases that can influence the final output and downstream analysis, presenting a challenge to comparability and reproducibility across studies [29, 30]. This underscores the importance of developing data workflows to maintain standardized data, ensure consistent results, and facilitate the reproducibility of findings across different studies.

Recently, several pipelines and workflows have been developed for microbiome research, although most are tailored to specific analyses and lack a holistic approach for broader data exploration. Additionally, many of these workflows do not offer detailed reports, flexible parameter adjustments, or meaningful downstream visualization options, which are essential for interpretation and reproducibility. For instance, Sunbeam focuses on some basic analyses, from data preprocessing and read‐based analysis to assembly‐based analysis [30]. The MetaSAMS workflow is primarily designed for taxonomic and functional analysis (read‐based analysis) [31], while MEGAnnotator2 [15], SqueezeMeta [32], and ATLAS [33] workflows were sequentially developed to reconstruct MAGs from metagenomic sequences (assembly‐based and binning). METABOLIC specifically profiles the functional traits of MAGs [34] and related workflows. In addition, online platforms, such as MicrobiomeAnalyst 2.0 [35] and Wekemo Bioincloud [36], offer useful tools for statistical meta‐analysis and shotgun data matrix profiling. However, these platforms struggle to process large datasets online, which is especially limiting for shotgun metagenomics, where data sizes often range from hundreds of gigabytes (GB) to several terabytes (TB).

Here, we introduce EasyMetagenome, an easily deployable and configurable pipeline that supports read‐based, assembly‐based, and binning analyses, as well as in‐depth prokaryote genome analysis, generating publication‐ready visualizations. EasyMetagenome includes four main components: software and database installation, data analysis pipeline, statistics and visualization, and a dedicated questions and answers section. Nearly all steps are configurable, with reasonable default settings that allow for rapid deployment without extensive parameter tuning. Additionally, detailed parameter explanations are provided, making it adaptable to various data scenarios.

## RESULT

### Overview of EasyMetagenome

**Figure 1:**
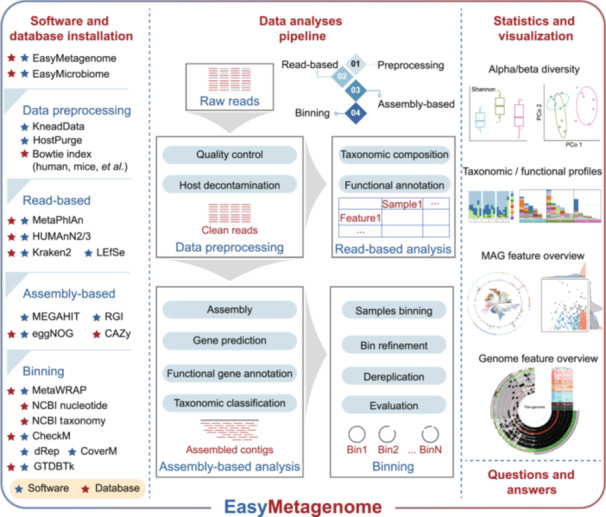
EasyMetagenome workflow. The workflow contains four main components, including software and database installation, data analysis pipeline, statistics and visualization, and the section for questions and answers.

Easymetagenome consists of four main components, software and database installation, the data analysis pipeline, statistics and visualization, and a section with dedicated questions and answers. Each component is a separate shell script, with a built‐in question and answer feature to help users understand and modify the process (Figure 1). It currently offers a portable workflow for data preprocessing, read‐based analysis, assembly‐based analysis, and binning, utilizing a range of state‐of‐the‐art software and data resources in the field.

After following our installation instructions for the necessary software and databases, users can input paired‐end metagenomic sequencing files to start the analysis. EasyMetagenome is a fully integrated, end‐to‐end pipeline that processes from raw reads to final data tables and publication‐ready figures (Figure 1). First, EasyMetagenome produces quality control and host genome decontamination of raw sequences/reads. Then, the clean reads can be analyzed for taxonomic composition and functional annotation through read‐based analysis, or undergo assembly, gene prediction, functional gene annotation, and taxonomic classification in assembly‐based analysis. In addition, the assembly is subsequently binned and refined using metaWARP, followed by dereplication and evaluation of recovered MAGs. For the statistics and visualization part, we have also provided various corresponding R configuration files, making it easy for users to generate and customize charts. As of November 27, 2024, EasyMetagenome has received 351 stars and 225 forks on Github (https://github.com/YongxinLiu/EasyMetagenome).

### Software installation and libraries database deployment

**Figure 2:**
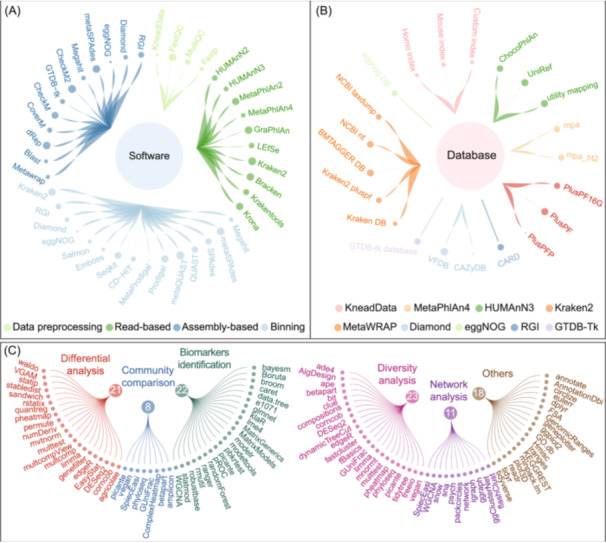
Software, databases, and R packages included in EasyMetagenome. (A) Software used in the EasyMetagenome pipeline, covering data pre‐processing, read‐based, assembly‐based, and binning analyses. (B) Standards or custom databases associated with each software. (C) R packages are utilized for differential analysis, community comparison, biomarker identification, diversity analysis, network analysis, and others.

The EasyMetagenome installation creates a bioinformatics environment that includes over 150 commonly used bioinformatics software and libraries, featuring 41 software, 23 databases, and more than 90 core R packages (Figure 2). To simplify the setup process, we provide at least two installation methods for each software, reducing the efforts required by researchers and freeing up a significant amount of their time to tackle more urgent tasks. The pipeline now includes four tools for data preprocessing, 10 tools for read‐based analysis, 15 tools for assembly‐based analysis, and 12 tools for binning (Figure 2A, Table 1).

**Table 1:** Open access tools that can be applied at each stage of metagenomic data.

Tool	Task	Repository
fastp v0.24.0	Short read quality control, filtering, and adapter removal	https://github.com/OpenGene/fastp
KneadData v0.12.0	Removal of host contamination	https://huttenhower.sph.harvard.edu/kneaddata
FastQC v0.12.1	Quality assessment of short reads	https://www.bioinformatics.babraham.ac.uk/projects/fastqc/
MetaPhlAn4 v4.0.6	Taxonomic profiling and relative abundance estimation	https://github.com/biobakery/MetaPhlAn
HUMAnN3 v3.7	Functional potential profiling of microbial communities	https://github.com/biobakery/humann
Kraken2 v2.1.3	Taxonomic classification	https://github.com/DerrickWood/kraken2
MEGAHIT v1.2.9	Short read assembly	https://github.com/voutcn/megahit
QUAST v5.0.2	Assembly quality assessment	https://github.com/ablab/quast
MetaProdigal v2.6.3	Gene prediction from assembled contigs	https://github.com/hyattpd/Prodigal
CD‐HIT v4.8.1	Clustering of assembled contigs	https://github.com/weizhongli/cdhit
Salmon v1.8.0	Contig coverage estimation	https://github.com/samtools/samtools
eggNOG v5.0	Protein functional annotation	https://github.com/eggnogdb/eggnog-mapper
MetaWRAP v1.3.2	Bin refinement and reassembly	https://github.com/bxlab/metaWRAP
dRep v2.6.2	Genome redundancy and representative genome selection	https://github.com/MrOlm/drep
CoverM v0.6.1	Coverage analysis of metagenomic assemblies	https://github.com/wwood/CoverM
GTDB‐tk v2.4.0	Taxonomic classification based on the genome taxonomy database	https://github.com/Ecogenomics/GTDBTk
CheckM2 v1.0.1	Quality control of microbial genome bins	https://github.com/chklovski/CheckM2
eggnog‐mapper v2.1.6	Functional annotation of genes using eggNOG database	https://github.com/eggnogdb/eggnog-mapper
DIAMOND v2.0.13	Fast protein sequence alignment	https://github.com/bbuchfink/diamond
RGI v5.2.0	Antibiotic resistance gene identification	https://github.com/arpcard/rgi
Bakta v1.9.4	Bacterial genome annotation	https://github.com/oschwengers/bakta
anvi'o v8	Pan‐genomic analysis of microbial genomes	https://github.com/merenlab/anvio
fastANI v1.34	Fast pairwise genome sequence comparison	https://github.com/ParBLiSS/FastANI

For commonly used public databases, we offer at least two accessible backup links (such as ftp server and Baidu NetDisk). Additionally, we provide detailed instructions for customizing database creation. For instance, we not only supply pre‐built indexing databases for mice (C57BL/6NJ) and humans (CRCh37) but also provide step‐by‐step guidance for constructing custom indexing databases using Bowtie2. To help users choose the appropriate database based on their server memory and usage needs, we offer three commonly used Kraken2 databases: a 16 GB version of standard database plus with protozoa and fungi (PlusPF16G), a 69 GB version of standard database plus with protozoa and fungi (PlusPF), and a 144 GB version of standard database plus with protozoa, fungi, and plant (PlusPFP). We also provide the guidance on building custom microbial databases (Figure 2B).

After that, we offer a range of R scripts for generating publication‐ready figures across various domains (Figure 2C). For difference analysis, we use 21 R packages to detect significant differences and visualize results. Diversity analysis is supported by 23 R packages, addressing alpha diversity, beta diversity, rarefaction, and compositional analysis. Community comparison employs eight key packages for ordination, clustering, and diversity metrics. Biomarker identification involves 22 packages utilizing machine learning and statistical modeling methods. Network analysis relies on 11 packages to identify and visualize relationships within datasets. Additionally, 18 packages assist with data manipulation, visualization, and annotation, ensuring a comprehensive analytical approach.

### Data analysis pipeline design

**Figure 3:**
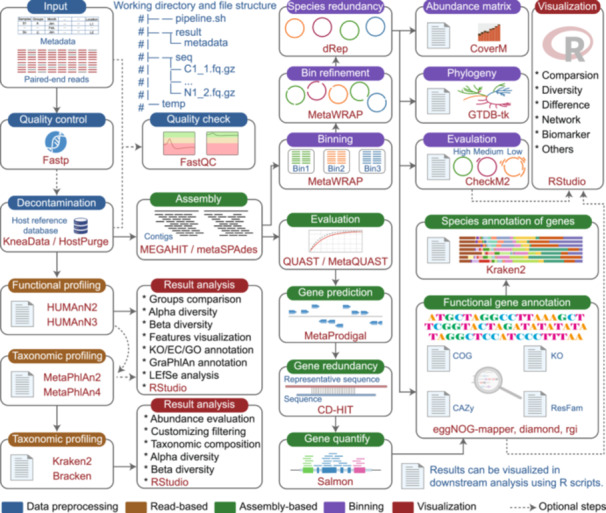
Summary of the EasyMetagenome analysis pipeline. EasyMetagenome pipeline includes data preprocessing for read‐based, assembly‐based, and binning analyses, with files available for data visualization. Dotted lines denote optional steps, and software are highlighted in red.

To guide the analysis process, we provide a clear working directory and file structure within our pipeline (Figure 3). The first step is to preprocess raw sequence data, which is essential for removing low‐quality reads and contamination from host‐associated sequences, ensuring cleaner and more reliable data for downstream analysis. The second step involves profiling the taxonomy and metabolic potential using a read‐based approach, which is crucial for identifying the microbial composition and functional capabilities, providing valuable insights into the ecosystem being studied. In this step, Kraken2 and HUMAnN2 or HUMAnN3 are used for microbial composition and functional analysis, respectively. This method is straightforward and widely used, while it may overlook many previously unknown genes that are not annotated in current databases.

The assembly step is a critical part of the process, where clean reads are assembled into contigs using tools like MEGAHIT or metaSPAdes. MEGAHIT is optimized for quickly assembling large, complex metagenomic datasets with minimal computational resources, while metaSPAdes produces longer and strain‐level contigs but requires more computational resources. Once the contigs are assembled, they can undergo assembly evaluation, gene prediction, removal of redundant genes, and gene abundance quantification. Metagenomic datasets typically contain millions of genes. Then these genes can be combined into functional categories, such as KEGG Orthology (KO), Clusters of Orthologous Genes (COG), Carbohydrate Active Enzyme (CAZy), Antibiotic Resistance Genes (ARG).

In the metagenome binning step, contigs are clustered into genome bins. This can be done individually for each set of assembled contigs or by pooling contigs from multiple samples and mapping each sample back to a shared catalog of contigs. Tools like metaWRAP facilitate this process, generating MAGs through contigs binning. In metagenomic studies, binning enables the reconstruction of both known and unknown genomes, providing a comprehensive description of the microbial community and serving as a foundation for further organismal analysis.

### Taxonomic and functional composition of read‐based analysis

**Figure 4:**
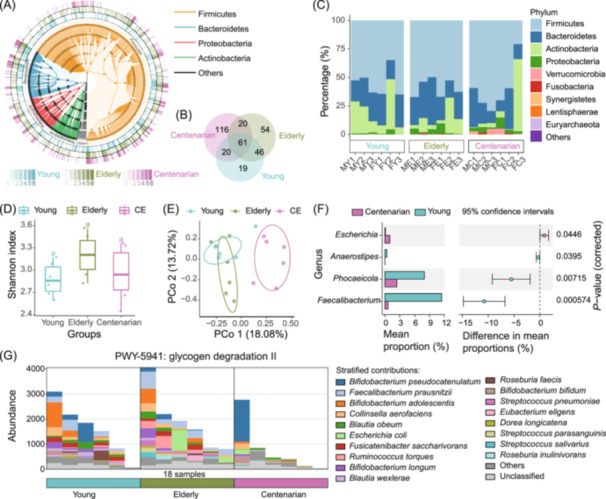
Taxonomic and functional composition from read‐based analysis using EasyMetagenome in human gut microbiome samples. (A) Microbial taxonomic composition overview across groups. The inner line color represents the Phylum. The outer bar shading represents taxa presence or absence across six representative samples per group. (B) Venn diagram showing the unique and shared taxa across three groups. (C) Taxonomic composition across groups and samples in phylum‐level. (D) Shannon diversity indices across groups, with letters indicating significant group differences (*p* ≤ 0.05, ANOVA, Tukey's HSD). (E) Principal coordinates analysis (PCoA) based on Bray‐Curtis dissimilarity (*p* < 0.001, PERMANOVA with ADONIS test). (F) STAMP plot showing genus‐level differences between young and centenarian groups. (G) Abundance of the functional pathway PWY‐5941 across groups and samples, including stratified contributions from specific microbial species. (A–F) are derived from species annotations with MetaPhlAn4. (G) represents functional potential analysis via HUMAnN3. The raw data from the Bioproject (PRJNA675598) of NCBI with accession number in Table S1.

Here, we present the results with read‐based analysis, showing the diversity of taxonomic and functional composition using data from centenarian's gut microbiome studies. Taxonomic composition is assessed using Kraken2 (Figure S1) and MetaPhlAn4 (Figure 4), followed by alpha and beta diversity analyses and functional composition assessment using HUMAnN3 (Figure 4). We provide multiple visualization styles to clarify and enrich the interpretation of these results, offering a detailed, multidimensional perspective on community structure and functional dynamics (Figure 4).

For taxonomic annotation of metagenomic data, both Kraken2 and MetaPhlAn4 offer distinct yet complementary methods. Kraken2 classifies taxa using a *k*‐mer‐based approach compatible with databases such as PlusPF16G, PlusPF, and PlusPFP (Figure 2B). To refine taxonomic abundance estimates, Bracken builds on Kraken2's results by redistributing reads probabilistically. MetaPhlAn4, alternatively, achieves taxonomic profiling using a clade‐specific marker gene catalog (e.g., mpa, mpa_bt2), offering a focused, gene‐centered approach to taxonomic annotation. For functional potential analysis, HUMAnN3 maps functional genes and pathways using resources like UniRef, ChocoPhlAn, and a set of utility mapping files.

To visualize species and functional composition, we profile bacterial distributions at the phylum level across centenarian, elderly, and young groups, highlighting species presence and relative abundance within each sample (Figure 4A). Venn diagrams illustrate shared and unique taxa among the groups (Figure 4B), while stacked bar charts compare taxa distributions between and within groups (Figure 4C). We calculate alpha diversity metrics, including alpha diversity indices (Chao1, ACE, Shannon, Simpson, Inverse Simpson), and beta diversity metrics (Bray‐Curtis, Euclidean, Jaccard, Manhattan) across samples and groups (Figure 4D,E). To analyze species differences between groups, STAMP plot is used, allowing groupwise comparisons with bar plots to illustrate the abundance of taxonomic unit or functional pathways (Figure 4F). Stratified contributions of these pathways by microbial species further illuminate community composition and function (Figure 4G). And the result of Kraken2 has been also displayed (Figure S1A,B).

### Functional annotation of genes from assembled contigs and comparative analysis of MAGs

**Figure 5:**
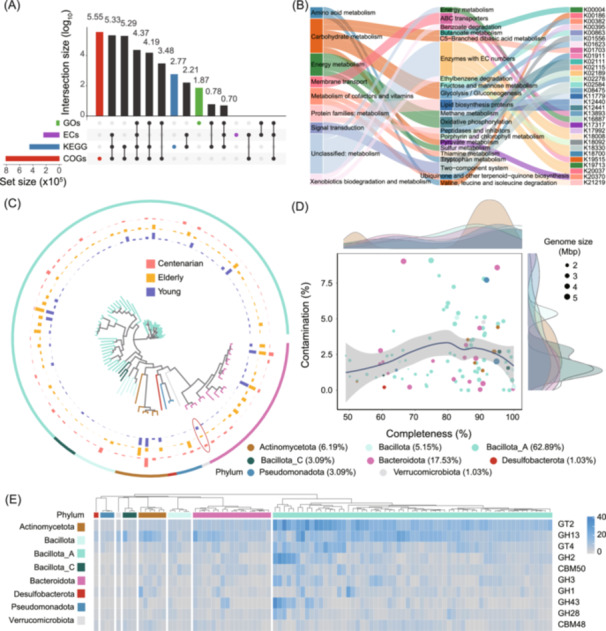
Functional annotation of assembled contigs and comparative analysis of MAGs in human gut microbiome samples. (A) Functional annotations of assembled contigs across four categories, showing the number of annotated proteins per category and their overlaps. Vertical bars represent unique (colored) or shared (black) proteins between categories; horizontal bars display the total protein count with functional annotations for each category. (B) KEGG classification of annotated functional genes at primary, secondary, and tertiary levels, visualized in a flowchart of functional distribution. (C) Phylogenetic analysis and distribution of metagenome‐assembled genomes (MAGs) across different groups, highlighting evolutionary relationships of representative MAGs. The tree colored by phylum. The bar shows mean relative abundance of MAGs in different groups calculated by CoverM. (D) The distribution of completeness and contamination on MAGs and the color of point represents phylum. (E) CAZy functional gene annotations within MAGs, illustrating the distribution of key carbohydrate‐active genes across MAGs.

Our assembly‐based analysis enables the functional annotation of gene sequences within assembled contigs using a range of comprehensive databases, including Gene Ontologies (GOs), level‐4 Enzyme Commission categories (ECs), Clusters of Orthologous Genes (COGs), carbohydrate‐active enzymes (CAZy), and Kyoto Encyclopedia of Genes and Genomes (KEGG) modules (Figure 5A, Figure S2A). This pipeline provides several key functionalities. First, it displays the distribution of protein functional annotations across categories and visualizes their overlap (Figure 5A). It also allows for the comparison of functional module abundance and diversity, including COGs, KOs, and CAZy, across distinct groups to explore microbiome functional variations (Figure S2A). Additionally, annotated functional genes are categorized into hierarchical levels (like KEGG), to show a structured distribution of functional roles across these levels (Figure 5B).

Following the binning pipeline, we can generate quality evaluations, species annotations, and functional profiles of MAGs. The binned MAGs can then be analyzed for mean coverage, phylogenetic inference, and species distribution across various taxonomic levels, displaying mean coverage across all samples (Figure S2B) or within specific groups (Figure 5C). The phyla information of MAGs is summarized with GTDB‐tk (Figure 5D, Figure S2C), and the completeness and contamination of MAGs are assessed in the CheckM (Figure S3). The high‐quality MAGs, with completeness ≥90% and contamination ≤5%, can be selected for further analysis to assess taxonomy, functional profiles, and the presence of CAZy (Figure 5E) or antibiotics resistance genes (Figure S2D).

### Characterization of *Alistipes putredinis* and pan‐genome analysis on MAGs

**Figure 6:**
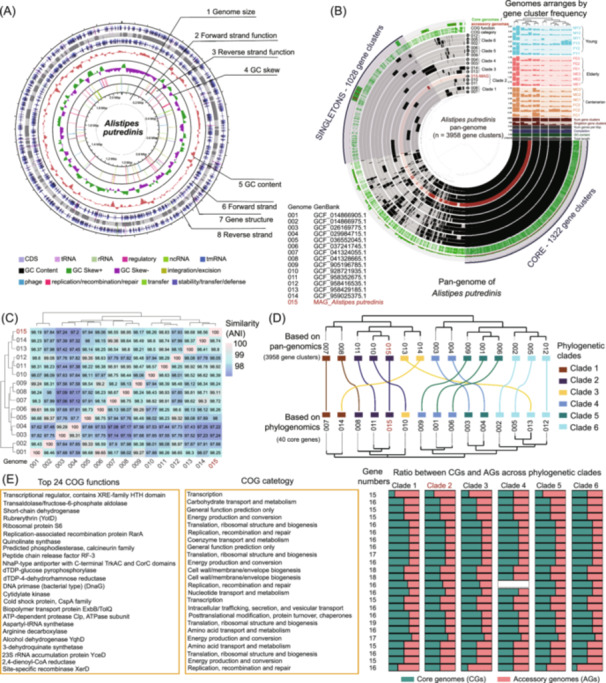
Metagenomic assembly genome digging in example human gut microbiome. (A) Genome map of *Alistipes putredinis* (ncRNA, COG annotation, GC content, and GC skew). From inner to outer, the 1st circle shows the genome size (1 Genome size); the 2nd circle shows the COG function of the forward strand gene, and each color represents a function classification (2 Forward strand function); the 3rd circle shows the function of the reverse strand gene (3 Reverse strand function); the 4th circle shows the GC skew ((G − C)/(G + C), green > 0, purple < 0) (4 GC skew); the 5th circle shows the GC content (5 GC content); the 6th circle shows the coding regions and structural RNA genes of the forward strand (6 Forward strand); the 7th circle shows the gene structure (7 Gene structure); the 8th circle shows the coding regions and structural RNA genes of the reverse strand (8 Reverse strand); (B) Pan‐genomic analysis of *Alistipes putredinis* genomes. Pan‐genome graph generated with Anvi'o, where the gene clusters (radial bars) are ordered by gene cluster frequency according to the organization of genes in the genome of *Alistipes putredinis*. And we divided 15 genomes of *Alistipes putredinis* used here into six clades. The circles show, from inner to outer, for the detected (black areas) or undetected (grey areas) of genes in each genome (circles 1‐15 with 6 clades arranged); we also mapping the gene clusters to the cluster of COG database, and illustrated COG category (circle 16) and COG functions annotated (circle 17) for gene clusters in which at least one gene was functionally annotated. In the outermost circle describes the ratio of core genomes and accessory genomes. Extending off the pan‐genome are bar plots showing the relevant properties for each genome. Above the genome property summaries, genome's median coverage across all centenarian (color in light brown), elderly (color in red), and young (color in cyan) pan‐genome samples are shown in bar graph separately. (C) Average nucleotide identity (ANI) mean heatmap generated using the fastANI tool depicts the genomic relatedness resulting from a multi‐genome comparison among strains of *Alistipes putredinis*. (D) Organization of *Alistipes putredinis* genomes based on gene clusters compared to phylogenomics. The cluster tree on the top showed the clustering of 15 genomes of *Alistipes putredinis* based on the 3958 gene clusters recovered from the pan‐genomic analysis. The bottom tree clustering the same genomes based on phylogenomics using 40 core genes. Colors showed six phylogenetic clades based on pan‐genomics. (E) The 24 most accessory functions identified in *Alistipes putredinis* isolates defined by high ratio of accessory genomes. The left two panels showed the COG functions and COG categories. The stack bar on the right showed an example ratio between core genomes (green) and accessory genomes (red) in different phylogenetic clades.

Our pipeline provides detailed functional annotation and analysis of isolates or MAGs. In this study, we focused on *Alistipes putredinis*, a prevalent species in the human gut microbiota, to gain in‐depth functional insights into this organism. The MAG of *Alistipes putredinis* was primarily identified in the centenarian group, showing 96.23% completeness, 0.67% contamination, a genome size of 2.08 Mb, and a GC content of 55% (Figure 6A).

To examine the shared and unique functional gene content of *Alistipes putredinis* MAG in comparison with 14 related *Alistipes putredinis* genomes, we conducted a pan‐genomic analysis (Figure 6B). This analysis identified a total of 3958 genes across the *Alistipes putredinis* pan‐genome, encompassing 14 publicly available *Alistipes putredinis* genomes and *Alistipes putredinis* MAG annotated from our 18 metagenome samples. Based on gene cluster frequency, these 15 genomes were divided into 6 clades, revealing 1322 core genes (33.4%) and 1028 singleton genes (26.0%) unique to individual genomes. Clustering the 15 genomes according to gene cluster frequency (both number and identity) further segregated them into two major groups and 6 clades. Each genome contained between 1821 and 2323 genes (top right bar chart), with core genes comprising 57%–73% of the total genes in each genome (Figure 6B).

Then we calculated the average nucleotide identity (ANI) among the 15 *Alistipes putredinis*, and found that *Alistipes putredinis* MAG shares a 97.22%–99.11% similarity with other published genomes (Figure 6C). Since shared gene content between genomes effectively predicts phylogenetic relationships, we used the distribution of genes to infer the relationships among these genomes. Compared with dendrograms generated using 40 core genes, the clustering of clades showed a notable difference (Figure 6D), suggesting that incorporating the whole genomic content, rather than only core genes, may enhance accuracy in determining relationships among different genomes. Our results may also suggest that using the whole genomic content instead of part of core genes might be advantageous to infer the relationship between different genomes. Finally, we also annotated the *Alistipes putredinis* genomes with functional data from the COG database. We selected 24 COG functions with high AG rates to demonstrate functional diversity within the pan‐genome. This allowed us to observe variations in CG/AG ratios across different clades for each selected COG function, providing insights into functional divergence among clades (Figure 6E).

## DISCUSSION

In EasyMetagenome, we provide several options for handling diverse shotgun metagenomic data, suitable for various scenarios. Facing the challenge of host contamination in metagenomic data [37, 38, 39], we developed HostPurge (https://github.com/HaoLuo-leo/HostPurge), a new tool that integrates popular alignment and *k*‐mer based methods, designed to enhance the efficiency, reliability, and comparability of large‐scale data mining. However, existing tools are limited when handling short‐read high‐throughput sequencing data without specific host reference genomes [39]. Artificial intelligence algorithms and third‐generation long‐read sequencing may help overcome this challenge, serving as a key to potentially establishing high‐quality MAGs databases. This highlights the urgent need to incorporate new algorithms and workflows tailored to diverse metagenomic data into our pipeline.

EasyMetagenome is primarily designed to run on Linux‐based systems, leveraging its robust support for high‐performance computational tools commonly used in metagenomic analysis. However, this focus may limit accessibility for users reliant on other operating systems, such as Windows or macOS. While compatibility can be addressed through virtualization or containerized solutions (e.g., Docker), these approaches may introduce additional complexity for nonspecialist users. Also, the scalability of the pipeline may be challenged when applied to datasets of exceptionally large size or complexity. Additionally, computational compatibility could vary depending on the specific hardware and software environments, particularly for users with limited access to high‐performance computing resources. Future iterations of EasyMetagenome could explore enhanced cross‐platform support to broaden its usability and accessibility.

The sequencing data produced by these third‐generation platforms differ qualitatively from second‐generation sequencing, necessitating customized analysis tools [40]. Although some bioinformatics software and methods have been developed for long‐read sequencing data, such as error correction methods [41] or hybrid assembly correction methods [42], the metagenomic analysis on third‐generation sequencing data is still in its early stages. Short‐read‐based algorithms for third‐generation metagenomic analysis are often not suitable for long‐read datasets under default parameters [43], which may lead to higher error rates when interpreting long sequencing reads. Therefore, there is an urgent need to establish a “gold standard” pipeline for third‐generation metagenomic data analysis and further enhance the comparability and reproducibility of microbiome exploration.

In the future, EasyMetagenome will focus on downstream data mining, using new methods to deepen our understanding of metagenomic data. For instance, network analysis has proven effective for exploring microbial interactions [44, 45], and identifying health‐relevant gut microbiota by revealing stable and functional genome relationships. A recent model, “two competing guilds,” uses abundance network analysis to identify core microbiome features as health indicators [44]. Another approach, generalized microbe phenotype triangulation, hypothesizes that in most paired expressions, microbial abundance strongly correlates (positively or negatively) with disease severity, which can assist in species and functional predictions. This method goes beyond traditional differential abundance approaches by targeting core microbiota directly associated with disease mechanisms and has been successfully applied in diversity research [46, 47]. With these advances, EasyMetagenome aims to enhance our understanding of microbial functions, interactions, and coevolution with hosts.

## CONCLUSION

As the demand for reproducibility in computational analysis of shotgun metagenomic data continues to increase, establishing a standardized bioinformatics workflow has become essential. To address this, we developed EasyMetagenome, a user‐friendly, flexible pipeline that supports comprehensive data exploration and provides clear parameter explanations at each step, generating publication‐ready results. In this study, we specifically present two examples to demonstrate the flexibility, usability, and reliability of our pipeline. This holistic pipeline to shotgun metagenomic analysis in microbiome research allows researchers to focus on resolving key questions in biological science rather than on navigating complex computational challenges.

Looking forward, we aim to address issues related to host contamination that impact data accuracy and processing efficiency. We will also integrate workflows optimized for third‐generation sequencing data and incorporate emerging algorithms, such as deep learning for cleaner microbiome reads, network analysis for interaction studies, and GMPT for key biomarker identification. Our goal is to enhance explainability and improve efficiency in microbiome data analysis.

## METHODS

### Workflow overview and software of EasyMetagenome

Our pipeline starts with Illumina raw sequencing reads, followed by data preprocessing, read‐based analysis, assembly‐based analysis, and binning. The resulting data is then statistically analyzed and visualized using R scripts.

Briefly, quality assessment can be performed using FastQC (https://www.bioinformatics.babraham.ac.uk/projects/fastqc/). Illumina raw paired‐end sequences are quality‐controlled using the fastp [18] software. The host sequence is then removed using KneadData (https://huttenhower.sph.harvard.edu/kneaddata) to generate the clean microbiome reads. After that, clean reads are rechecked for quality with FastQC. Taxonomic classification is carried out using Kraken2 [21], and species abundance is estimated. Gene family and pathway relative abundances are determined with HUMAnN2 or HUMAnN3 [23].

Concurrently, clean reads are assembled with either MEGAHIT [25] or metaSPAdes [26]. Subsequently, the assembled contigs are analyzed in two main streams. For assembly‐based analysis, contig quality is evaluated using QUAST [48]. Gene prediction is performed with MetaProdigal [49], gene redundancy is assessed using CD‐HIT [50], and gene quantification is carried out with Salmon [51]. The Kyoto Encyclopedia of Genes and Genomes (KEGG) Orthology (KO) profiles [52], Carbohydrate Active Enzyme (CAZy) [53], and Clusters of Orthologous Groups of proteins (COG) [54] database are used for microbiome functional annotation using eggNOG [55]. The antibiotic resistance genes were also detected using the ResFams database [56] with RGI [57]. And Kraken2 [21] is used for species annotation of genes.

For binning, MAGs are retrieved using the “binning” and “bin_refinement” modules in MetaWRAP [25]. Species redundancy is addressed with dRep [58], abundance matrices are created with CoverM, phylogeny is determined using GTDB‐tk [59], and completeness and contamination of the bins are estimated with CheckM2 [60]. Functional gene annotation of MAGs is again performed using eggnog‐mapper [61], DIAMOND [62], and RGI [57].

### Data set selection and quality control for EasyMetagenome analysis

To showcase the functionality of EasyMetagenome, we analyzed datasets from previously published data, including a set of human gut microbiome samples [63] and a set of environmental microbiome samples [64], highlighting the pipeline's versatility and visualization capabilities across diverse sample types. For human gut samples, it contains three groups of Young (23–47 years old), Elderly (85–89 years old), and Centenarian (100+ years old), each represented by six samples with an equal number of male and female people (Table S1). For environmental samples, we selected four composting phases (Cooling, Mature, Mesophilic, and Thermophilic), each with four samples for replication (Table S2). Detailed accession numbers for these datasets are available in Tables S1 and S2 on NCBI.

All raw data were quality‐controlled with fastp v0.24.0 [18], followed by KneadData v0.12.0 to isolate high‐quality microbial reads [65], with human reads mapped to GRCh37 human reference genome and removed using Bowtie2 [20] (v2.5.1; parameters: ‐‐very‐sensitive ‐‐dovetail). The cleaned reads were then assessed for quality using FastQC v0.12.1. The clean microbial reads were analyzed using three complementary approaches—read‐based, assembly‐based, and binning analysis—demonstrating the EasyMetagenome pipeline's ability to generate publication‐quality visualizations.

### Read‐based analysis of EasyMetagenome in published metagenomic data

For read‐based analysis, high‐quality microbial reads were used for species‐level community profiling, with relative abundances determined using MetaPhlAn4 v4.0.6 [66], applying against the mpa_vOct22_CHOCOPhlAnSGB_202212 database under default parameters to capture all taxonomic levels. Additionally, functional potential profiling is performed using HUMAnN3 v3.7 [23]. Various EasyMetagenome scripts and dependent MicrobiomeStatPlot [67] were then applied to visualize these results: “metaphlan_hclust_heatmap.R” generated heatmaps showing taxonomic distribution at different levels across groups, while “graphlan_plot54.r” illustrated the overall taxonomic structures within groups. Additionally, “otu_mean.R” calculated the mean taxonomy between groups, “sp_vennDiagram.sh” created a Venn diagram, and “metaphlan_boxplot.R” produced boxplots displaying taxonomic levels across groups. For alpha diversity analysis, we used “otutab_rare.R” to calculate indices such as Richness, Chao1, ACE, Shannon, Simpson, and Inverse Simpson, and “alpha_boxplot.R” to generate boxplots for these metrics from EasyAmplicon [68, 69] ANOVA followed by Tukey's HSD test was applied to assess the significance of differences and determine the *p*‐value. For beta diversity analysis, “usearch10” was used to calculate Bray–Curtis, Euclidean, Jaccard, and Manhattan metrics, and the results were visualized as PCoA plots using “beta_pcoa.R”. PERMANOVA with the ADONIS test was employed to evaluate the statistical significance and compute the *p*‐value. The “tax_stackplot.R” script was used to display a stackplot of taxonomic composition across groups, while “compare_stamp.R” allowed for taxonomic comparisons between two groups. Additionally, “sp_pheatmap.sh” calculated pathway abundance differences, and “humann_barplot” of HUMAnN3 v3.7 [23] was used to visualize taxonomic composition within a specific pathway across groups.

Taxonomic classification was performed using Kraken2 v2.1.3 [21] with default settings and a pre‐built PlusPF database, while species abundance was estimated with Bracken v2.7 [22] to refine species‐level abundance estimates. Both Kraken2 and MetaPhlAn4 can be used for taxonomic profiling, but they employ distinct, yet complementary, methods and reference databases. Kraken2 utilizes a *k*‐mer‐based classification approach, comparing reads against a standard or user‐constructed database. In contrast, MetaPhlAn performs classification by aligning query reads to a database of clade‐specific marker genes [22]. To leverage the strengths of each, EasyMetagenome includes visualizations generated from both methods. For Kraken2 data, we converted its output format to match MetaPhlAn's structure, applying the same visualization techniques described above to produce taxonomic annotation images based on Kraken2 results.

### Assembly‐based analysis of EasyMetagenome in published metagenomic data

For assembly‐based analysis, high‐quality microbial reads were *de novo* assembled separately using MEGAHIT v1.2.9 [25], with assembly metrics subsequently assessed using QUAST v5.0.2 [48]. MetaProdigal v2.6.3 [49] was used for gene prediction, and assembled contigs were clustered using CD‐HIT v4.8.1 [48] with 95% identity and 90% coverage (‐aS 0.9 ‐c 0.95). Then Salmon v1.8.0 [51] was employed to measure the abundance of contigs in each sample. Protein functions, including KEGG Ortholog (KO) [52], CAZy [53], and COGs [54] were annotated using eggNOG‐mapper based on eggNOG v5.0 clusters [55]. To generate a summarized abundance table, “format_dbcan2list.pl” was used in combination with “summarizeAbundance.py”. Subsequently, R was used to visualize the functional annotations of genes across four categories, classify annotated functional genes at various hierarchical levels, and compare functional module levels of the microbiome across different groups.

### Binning the metagenome‐assembled genomes in published metagenomic data with the EasyMetagenome pipeline

For binning, MAGs were binned from contigs assembled with MEGAHIT v1.2.9 [25], using the “binning” and “bin_refinement” modules in MetaWRAP v1.3.2 [25]. In detail, contigs were clustered into metagenomic bins using the “binning” module (‐‐metabat2 ‐‐maxbin2), followed by refinement with the “bin_refinement” module (‐c 50 ‐x 10). After that, the resulting refined bins were dereplicated using dRep v2.6.2 [58], with the parameters “‐sa 0.95 ‐nc 0.3 ‐p 16 ‐comp 50 ‐con 10.” The abundance matrices of the dereplicated genomes across various samples were then evaluated using CoverM v0.6.1.

Taxonomic classification of the refined bins was performed using the “gtdbtk classify_wf” module from the GTDB‐tk v2.4.0 (r214) database, and their phylogenetic relationships were inferred with the “gydbtk infer” module in GTDB‐tk v2.4.0 [59]. The completeness and contamination of the MAGs were estimated with CheckM2 v1.0.1 [60], with high‐quality MAGs selected based on the following thresholds: High‐quality: ≥90% completeness and ≤5% contamination (for some environmental samples, adjustments may be required based on actual conditions); Medium‐quality: >50% completeness and ≤5% contamination; Low‐quality: <50% completeness. Subsequently, functional annotations of the high‐quality MAGs, including KEGG Ortholog (KO), CAZy, and COGs, were performed using eggnog‐mapper v2.1.6 [61], DIAMOND v2.0.13 [62], and RGI v5.2.0 [57]. Finally, R was used to visualize the genome quality of the MAGs, perform phylogenetic analysis, and display the distribution of MAGs, as well as the functional gene annotations within them.

### Genome structures and pan‐genomic analysis of *Alistipes putredinis*

After obtaining the MAG annotation results for all samples, we selected *Alistipes putredinis* from human gut metagenomic data (*Alistipes putredinis* MAG) for single bacterial genome annotation and pan‐genomic analysis due to its high completeness (96.23%) and low contamination rate (0.67%). For single bacterial genome annotation, we first used Bakta v1.9.4 to annotate the *Alistipes putredinis* MAG [70]. Once the genome annotation results were obtained, we imported the annotated files in “embl” format into Proksee software for further analysis and visualization [71]. We also calculated GC skew to assess the relative amounts of guanine (G) and cytosine (C) in the selected genome, and performed additional genome annotation of *Alistipes putredinis* using mobileOG‐db to identify mobile orthologous groups [72].

For pan‐genomic analysis, we used anvi'o v8 to analyze the assembled genomes of *Alistipes putredinis* [73], incorporating both the *Alistipes putredinis* MAG and 14 publicly available *Alistipes putredinis* isolates from NCBI (Table S3). These 15 *Alistipes putredinis* genomes were used to construct a contig database with the “anvi‐gen‐contigs‐database” function in anvi'o v8. Bacterial single‐copy gene information was then retrieved using the “anvi‐run‐hmms” function, and NCBI COG function annotations were obtained using the “anvi‐run‐ncbi‐cogs” function. We mapped short reads from human gut microbiome samples to the constructed contig database and sorted the recruited reads using Bowtie2 [20] and samtools [74], separately. After recruiting metagenomic short reads from different age groups, we used the “anvi‐profile” function to process the recruitment files and generate profile databases containing gene coverage and detection information for contig database. These profiles were merged into a single database for each sample, linking the contigs to their respective genomes using the “anvi‐import‐collection” function. Finally, we computed the pan‐genome of *Alistipes putredinis* isolates using the “anvi‐gen‐genomes‐storage” function for genome storage and the “anvi‐pan‐pangenome” function to generate the *Alistipes putredinis* pan‐genome. The pan‐genome was then visualized using the “anvi‐display‐pan” function in the anvi'o interactive interface [75].

After obtaining the pan‐genomes of *Alistipes putredinis*, we compared the clustering of *Alistipes putredinis* genomes based on all gene clusters detected in the pan‐genomic analysis with clustering based on phylogenomics, using 40 core genes selected from 1,322 core genes identified through the pan‐genomic analysis. To further assess genomic similarity, we used fastANI v1.34 [76] to calculate the average nucleotide identity (ANI) between the *Alistipes putredinis* MAG and the other 14 published *Alistipes putredinis* genomes. Additionally, to examine the functional composition of the *Alistipes putredinis* pan‐genomes, we displayed the most accessory functions (with unusually high AG ratios) identified across all *Alistipes putredinis* genomes using the COG database [54].

## AUTHOR CONTRIBUTIONS

**Defeng Bai**: Methodology; software; validation; writing—original draft; writing—review and editing. **Tong Chen**: Conceptualization; funding acquisition; methodology; software; validation; writing—original draft; writing—review and editing. **Jiani Xun**: Methodology; software; validation; writing—original draft; writing—review and editing. **Chuang Ma**: Methodology; software; validation; writing—original draft; writing—review and editing. **Hao Luo**: Methodology; software; validation; writing—original draft; writing—review and editing. **Haifei Yang**: Methodology; software; validation; writing—original draft; writing—review and editing. **Chen Cao**: Software; validation; writing—review and editing. **Xiaofeng Cao**: Software; validation; writing—review and editing. **Jianzhou Cui**: Software; validation; writing—review and editing. **Yuan‐Ping Deng**: Software; validation; writing—review and editing. **Zhaochao Deng**: Software; validation; writing—review and editing. **Wenxin Dong**: Software; validation; writing—review and editing. **Wenxue Dong**: Software; validation; writing—review and editing. **Juan Du**: Software; validation; writing—review and editing. **Qunkai Fang**: Software; validation; writing—review and editing. **Wei Fang**: Software; validation; writing—review and editing. **Yue Fang**: Software; validation; writing—review and editing. **Fangtian Fu**: Software; validation; writing—review and editing. **Min Fu**: Software; validation; writing—review and editing. **Yi‐Tian Fu**: Software; validation; writing—review and editing. **He Gao**: Software; validation; writing—review and editing. **Jingping Ge**: Software; validation; writing—review and editing. **Qinglong Gong**: Software; validation; writing—review and editing. **Lunda Gu**: Software; validation; writing—review and editing. **Peng Guo**: Software; validation; writing—review and editing. **Yuhao Guo**: Software; validation; writing—review and editing. **Tang Hai**: Software; validation; writing—review and editing. **Hao Liu**: Software; validation; writing—original draft. **Jieqiang He**: Software; validation; writing—original draft. **Zi‐Yang He**: Software; validation; writing—review and editing. **Huiyu Hou**: Software; validation; writing—review and editing. **Can Huang**: Software; validation; writing—review and editing. **Shuai Ji**: Software; validation; writing—original draft. **ChangHai Jiang**: Software; validation; writing—review and editing. **Gui‐Lai Jiang**: Software; validation; writing—review and editing. **Lingjuan Jiang**: Software; validation; writing—review and editing. **Ling N. Jin**: Software; validation; writing—review and editing. **Yuhe Kan**: Software; validation; writing—review and editing. **Da Kang**: Software; validation; writing—review and editing. **Jin Kou**: Software; validation; writing—review and editing. **Ka‐Lung LAM**: Software; validation; writing—original draft. **Changchao Li**: Software; validation; writing—original draft. **Chong Li**: Software; validation; writing—review and editing. **Fuyi Li**: Software; validation; writing—review and editing. **Liwei Li**: Software; validation; writing—review and editing. **Miao Li**: Software; validation; writing—review and editing. **Xin Li**: Software; validation; writing—review and editing. **Ye Li**: Software; validation; writing—review and editing. **Zheng‐Tao Li**: Software; validation; writing—review and editing. **Jing Liang**: Resources; validation; writing—review and editing. **Yongxin Lin**: Software; validation; writing—review and editing. **Changzhen Liu**: Software; validation; writing—review and editing. **Danni Liu**: Software; validation; writing—review and editing. **Fengqin Liu**: Software; validation; writing—review and editing. **Jia Liu**: Software; validation; writing—review and editing. **Tianrui Liu**: Software; validation; writing—review and editing. **Tingting Liu**: Software; validation; writing—review and editing. **Xinyuan Liu**: Software; validation; writing—review and editing. **Yaqun Liu**: Software; validation; writing—review and editing. **Bangyan Liu**: Software; validation; writing—original draft. **Minghao Liu**: Software; validation; writing—review and editing. **Wenbo Lou**: Software; validation; writing—review and editing. **Yaning Luan**: Software; validation; writing—review and editing. **Yuanyuan Luo**: Software; validation; writing—review and editing. **Hujie Lv**: Software; validation; writing—review and editing. **Tengfei Ma**: Software; validation; writing—review and editing. **Zongjiong Mai**: Software; validation; writing—review and editing. **Jiayuan Mo**: Software; validation; writing—review and editing. **Dongze Niu**: Software; validation; writing—review and editing. **Zhuo Pan**: Software; validation; writing—review and editing. **Heyuan Qi**: Software; validation; writing—review and editing. **Zhanyao Shi**: Software; validation; writing—review and editing. **Chunjiao Song**: Software; validation; writing—review and editing. **Fuxiang Sun**: Software; validation; writing—review and editing. **Yan Sun**: Software; validation; writing—review and editing. **Sihui Tian**: Software; validation; writing—review and editing. **Xiulin Wan**: Software; validation; writing—review and editing. **Guoliang Wang**: Software; validation; writing—review and editing. **Hongyang Wang**: Software; validation; writing—review and editing. **Hongyu Wang**: Software; validation; writing—review and editing. **Huanhuan Wang**: Software; validation; writing—review and editing. **Jing Wang**: Software; validation; writing—review and editing. **Jun Wang**: Software; validation; writing—review and editing. **Kang Wang**: Software; validation; writing—review and editing. **Leli Wang**: Software; validation; writing—review and editing. **Shao‐kun Wang**: Software; validation; writing—review and editing. **Xinlong Wang**: Software; validation; writing—review and editing. **Yao Wang**: Software; validation; writing—review and editing. **Zufei Xiao**: Software; validation; writing—review and editing. **Huichun Xing**: Software; validation; writing—review and editing. **Yifan Xu**: Software; validation; writing—review and editing. **Shu‐yan Yan**: Software; validation; writing—review and editing. **Li Yang**: Software; validation; writing—review and editing. **Song Yang**: Software; validation; writing—review and editing. **Yuanming Yang**: Software; validation; writing—review and editing. **Xiaofang Yao**: Software; validation; writing—review and editing. **Salsabeel Yousuf**: Software; validation; writing—review and editing. **Hao Yu**: Software; validation; writing—review and editing. **Yu Lei**: Software; validation; writing—review and editing. **Zhengrong Yuan**: Software; validation; writing—review and editing. **Meiyin Zeng**: Software; validation; writing—review and editing. **Chunfang Zhang**: Software; validation; writing—review and editing. **Chunge Zhang**: Software; validation; writing—review and editing. **Huimin Zhang**: Software; validation; writing—review and editing. **Jing Zhang**: Software; validation; writing—review and editing. **Na Zhang**: Software; validation; writing—review and editing. **Tianyuan Zhang**: Software; validation; writing—review and editing. **Yi‐Bo Zhang**: Software; validation; writing—review and editing. **Yupeng Zhang**: Software; validation; writing—review and editing. **Zheng Zhang**: Software; validation; writing—review and editing. **Mingda Zhou**: Software; validation; writing—review and editing. **Yuanping Zhou**: Software; validation; writing—review and editing. **Chengshuai Zhu**: Software; validation; writing—review and editing. **Lin Zhu**: Software; validation; writing—review and editing. **Yue Zhu**: Software; validation; writing—review and editing. **Zhihao Zhu**: Software; validation; writing—review and editing. **Hongqin Zou**: Software; validation; writing—review and editing. **Anna Zuo**: Software; validation; writing—review and editing. **Wenxuan Dong**: Software; validation; writing—review and editing. **Tao Wen**: Conceptualization; funding acquisition; validation; visualization; writing—original draft; writing—review and editing. **Shifu Chen**: Conceptualization; funding acquisition; validation; visualization; writing—original draft; writing—review and editing. **Guoliang Li**: Conceptualization; funding acquisition; validation; visualization; writing—original draft; writing—review and editing. **Yunyun Gao**: Conceptualization; funding acquisition; validation; visualization; writing—original draft; writing—review and editing. **Yong‐Xin Liu**: Conceptualization; funding acquisition; validation; visualization; writing—original draft; writing—review and editing.

## CONFLICT OF INTEREST STATEMENT

The authors declare no conflicts of interest.

## ETHICS STATEMENT

No ethics approval was required for this study because all samples used were publicly available and obtained from open‐access sources.

## Supporting information


**Figure S1.** Taxonomic profiling of environmental microbiome samples via EasyMetagenome read‐based analysis. (A) Taxonomic composition across groups (left) and samples (right) at the phylum level. (B) Alpha and beta diversity among groups. Alpha diversity analysis, including Shannon (left) and richness (middle) indices, with letters indicating significant differences between groups (*P* < 0.05, ANOVA, Tukey's HSD). Beta diversity (right) diversity analysis using principal coordinates analysis (PCoA) based on Bray‐Curtis dissimilarity (*P* < 0.001, PERMANOVA with ADONIS test).
**Figure S2.** Functional annotation of assembled contigs and comparative analysis of MAGs in environmental microbiome samples. (A) Comparison of the levels of functional modules (COGs, KOs, and CAZy) of the microbiome across different groups. The left panel shows sets included in the intersection and independent sites, and the right bar or pie charts show the categories of the functional modules in these sets. The major enriched categories are shown in the legend. (B) Phylogenetic analysis and distribution of metagenome assembled genomes (MAGs) across different groups, highlighting evolutionary relationships of representative MAGs. The bar shows mean coverage of MAGs in all samples calculated by CoverM. (C) The distribution of completeness and contamination in MAGs, with the color of the points representing the phylum. (D) Antibiotics resistance genes annotations within MAGs. The raw data from the Bioproject (PRJNA918803 and PRJNA917055) of NCBI with accession number in Table S2.
**Figure S3.** Genome quality of MAGs in environmental microbiome samples. (A) Completeness and contamination scores for all MAGs, colored by their quality classification categories. (B) Contigs N50 distribution for all MAGs. (C) GC content for all MAGs. High: ≥90% completeness, ≤5% contamination. Medium: >50% completeness, ≤5% contamination. Low: <50% completeness.

**Table S1.** Detailed information for human gut samples.
**Table S2.** Detailed information for environmental samples.
**Table S3.** Comparative average nucleotide identity (ANI) analysis between *Alistipes putredinis* MAG and diverse isolates of *Alistipes putredinis* from NCBI.

## Data Availability

EasyMetagenome is freely available, implemented in Shell and R, and is easy to install. Step‐by‐step protocols are provided on GitHub https://github.com/YongxinLiu/EasyMetagenome. All figure data and supplementary tables can be accessed via GitHub https://github.com/YongxinLiu/EasyMetagenome/tree/master/appendix. Supplementary materials (figures, tables, graphical abstract, slides, videos, Chinese translated version and update materials) may be found in the online DOI or iMeta Science http://www.imeta.science/.
