# Supplementary material for: EasyMetagenome: A user‐friendly and flexible pipeline for shotgun metagenomic analysis in microbiome research
Source: Imeta. 2025 Feb 14;4(1):e70001. doi: 10.1002/imt2.70001 (PMC11865343; doi:10.1002/imt2.70001)
Supplement: Supplementary file 1 — Figure S1. Taxonomic profiling of environmental microbiome samples via EasyMetagenome read‐based analysis. (A) Taxonomic composition across groups (left) and samples (right) at the phylum level. (B) Alpha and beta diversity among groups. Alpha diversity analysis, including Shannon (left) and richness (middle) indices, with letters indicating significant differences between groups (P < 0.05, ANOVA, Tukey's HSD). Beta diversity (right) diversity analysis using principal coordinates analysis (PCoA) based on Bray‐Curtis dissimilarity (P < 0.001, PERMANOVA with ADONIS test). Figure S2. Functional annotation of assembled contigs and comparative analysis of MAGs in environmental microbiome samples. (A) Comparison of the levels of functional modules (COGs, KOs, and CAZy) of the microbiome across different groups. The left panel shows sets included in the intersection and independent sites, and the right bar or pie charts show the categories of the functional modules in these sets. The major enriched categories are shown in the legend. (B) Phylogenetic analysis and distribution of metagenome assembled genomes (MAGs) across different groups, highlighting evolutionary relationships of representative MAGs. The bar shows mean coverage of MAGs in all samples calculated by CoverM. (C) The distribution of completeness and contamination in MAGs, with the color of the points representing the phylum. (D) Antibiotics resistance genes annotations within MAGs. The raw data from the Bioproject (PRJNA918803 and PRJNA917055) of NCBI with accession number in Table S2. Figure S3. Genome quality of MAGs in environmental microbiome samples. (A) Completeness and contamination scores for all MAGs, colored by their quality classification categories. (B) Contigs N50 distribution for all MAGs. (C) GC content for all MAGs. High: ≥90% completeness, ≤5% contamination. Medium: >50% completeness, ≤5% contamination. Low: <50% completeness. [file IMT2-4-e70001-s001.docx]

**Supporting information to**

**EasyMetagenome: A user-friendly and flexible pipeline for shotgun metagenomic analysis in microbiome research**

**Running title:** EasyMetagenome: A flexible pipeline for shotgun metagenomics

Defeng Bai^#^, Tong Chen^#*^, Jiani Xun^#^, Chuang Ma^#^, Hao Luo^#^, Haifei Yang^#^, Chen Cao, Xiaofeng Cao, Jianzhou Cui, Yuan-Ping Deng, Zhaochao Deng, Wenxin Dong, Wenxue Dong, Juan Du, Qunkai Fang, Wei Fang, Yue Fang, Fangtian Fu, Min Fu, Yi-Tian Fu, He Gao, Jingping Ge, Qinglong Gong, Lunda Gu, Peng Guo, Yuhao Guo, Tang Hai, Hao Liu, Jieqiang He, Zi-Yang He, Huiyu Hou, Can Huang, Shuai Ji, ChangHai Jiang, Gui-Lai Jiang, Lingjuan Jiang, Ling N. Jin, Yuhe Kan, Da Kang, Jin Kou, Ka-Lung LAM, Changchao Li, Chong Li, Fuyi Li, Liwei Li, Miao Li, Xin Li, Ye Li, Zheng-Tao Li, Jing Liang, Yongxin Lin, Changzhen Liu, Danni Liu , Fengqin Liu, Jia Liu, Tianrui Liu, Tingting Liu, Xinyuan Liu, Yaqun Liu, Bangyan Liu, Minghao Liu, Wenbo Lou, Yaning Luan, Yuanyuan Luo, Hujie Lv, Tengfei Ma, Zongjiong Mai, Jiayuan Mo, Dongze Niu, Zhuo Pan, Heyuan Qi, Zhanyao Shi, Chunjiao Song, Fuxiang Sun, Yan Sun, Sihui Tian, Xiulin Wan, Guoliang Wang, Hongyang Wang, Hongyu Wang, Huanhuan Wang, Jing Wang, Jun Wang, Kang Wang, Leli Wang, Shao-kun Wang, Xinlong Wang, Yao Wang, Zufei Xiao, Huichun Xing, Yifan Xu, Shu-yan Yan, Li Yang, Song Yang, Yuanming Yang, Xiaofang Yao, Salsabeel Yousuf, Hao Yu, Yu Lei, Zhengrong Yuan, Meiyin Zeng, Chunfang Zhang, Chunge Zhang, Huimin Zhang, Jing Zhang, Na Zhang, Tianyuan Zhang, Yi-Bo Zhang, Yupeng Zhang, Zheng Zhang, Mingda Zhou, Yuanping Zhou, Chengshuai Zhu, Lin Zhu, Yue Zhu, Zhihao Zhu, Hongqin Zou, Anna Zuo, Wenxuan Dong, Tao Wen^*^, Shifu Chen^*^, Guoliang Li^*^, Yunyun Gao^*^, Yong-Xin Liu^*^

Genome Analysis Laboratory of the Ministry of Agriculture and Rural Affairs, Agricultural Genomics Institute at Shenzhen, Chinese Academy of Agricultural Sciences, Shenzhen, Guangdong, China

^#^These authors contributed equally: Defeng Bai, Tong Chen, Jiani Xun, Chuang Ma, Hao Luo, Haifei Yang

^*^Correspondence: chentong_biology@163.com (Tong Chen); taowen@njau.edu.cn (Tao wen); chen@haplox.com (Shifu Chen); guoliangli2016@gmail.com (Guoliang Li); gaoyunyun@caas.cn (Yunyun Gao); liuyongxin@caas.cn (Yong-Xin Liu)

####
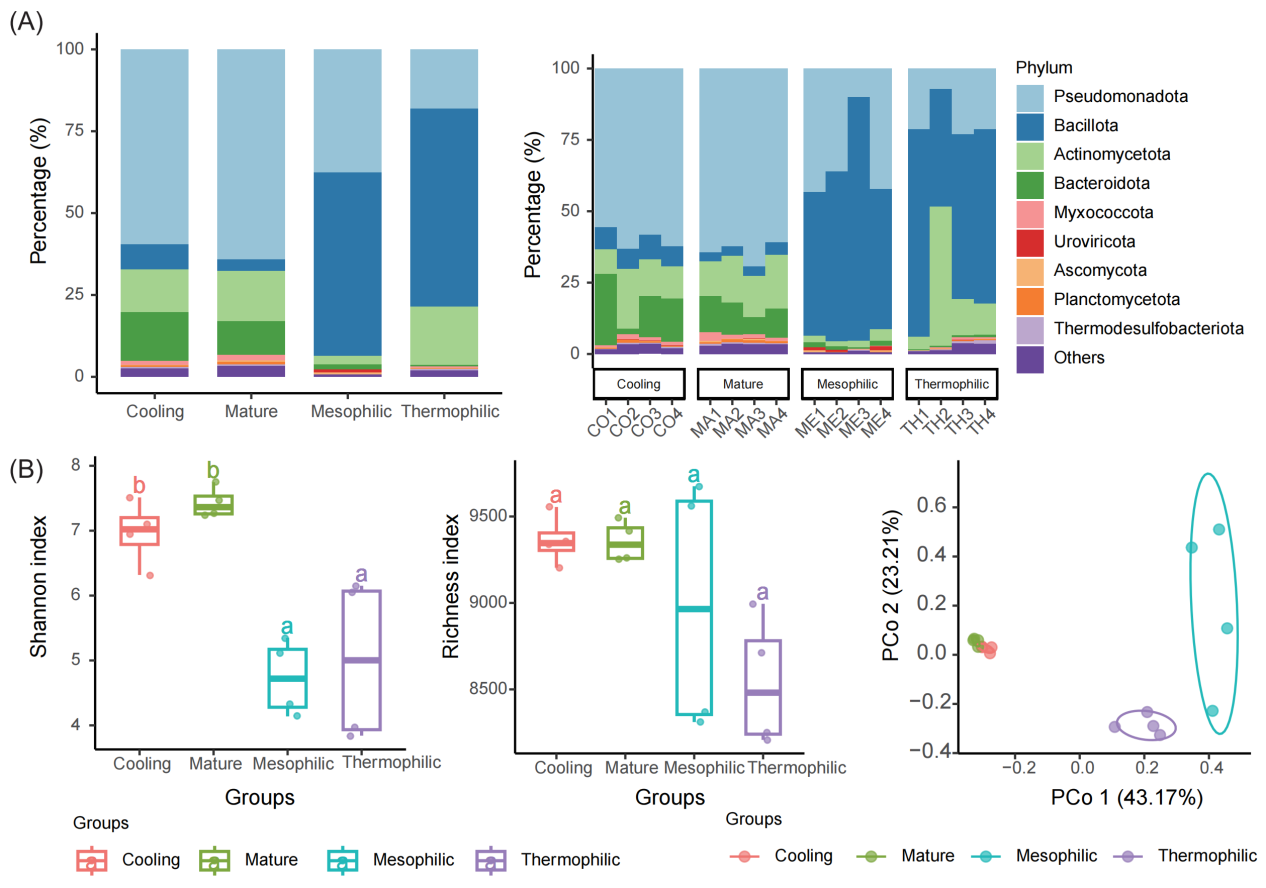


#### Figure S1. Taxonomic profiling of environmental microbiome samples via EasyMetagenome read-based analysis. (A). Taxonomic composition across groups (left) and samples (right) at the phylum level. (B) Alpha and beta diversity among groups. Alpha diversity analysis, including Shannon (left) and richness (middle) indices, with letters indicating significant differences between groups (*P* < 0.05, ANOVA, Tukey’s HSD). Beta diversity (right) diversity analysis using principal coordinates analysis (PCoA) based on Bray-Curtis dissimilarity (*P* < 0.001, PERMANOVA with ADONIS test).

####
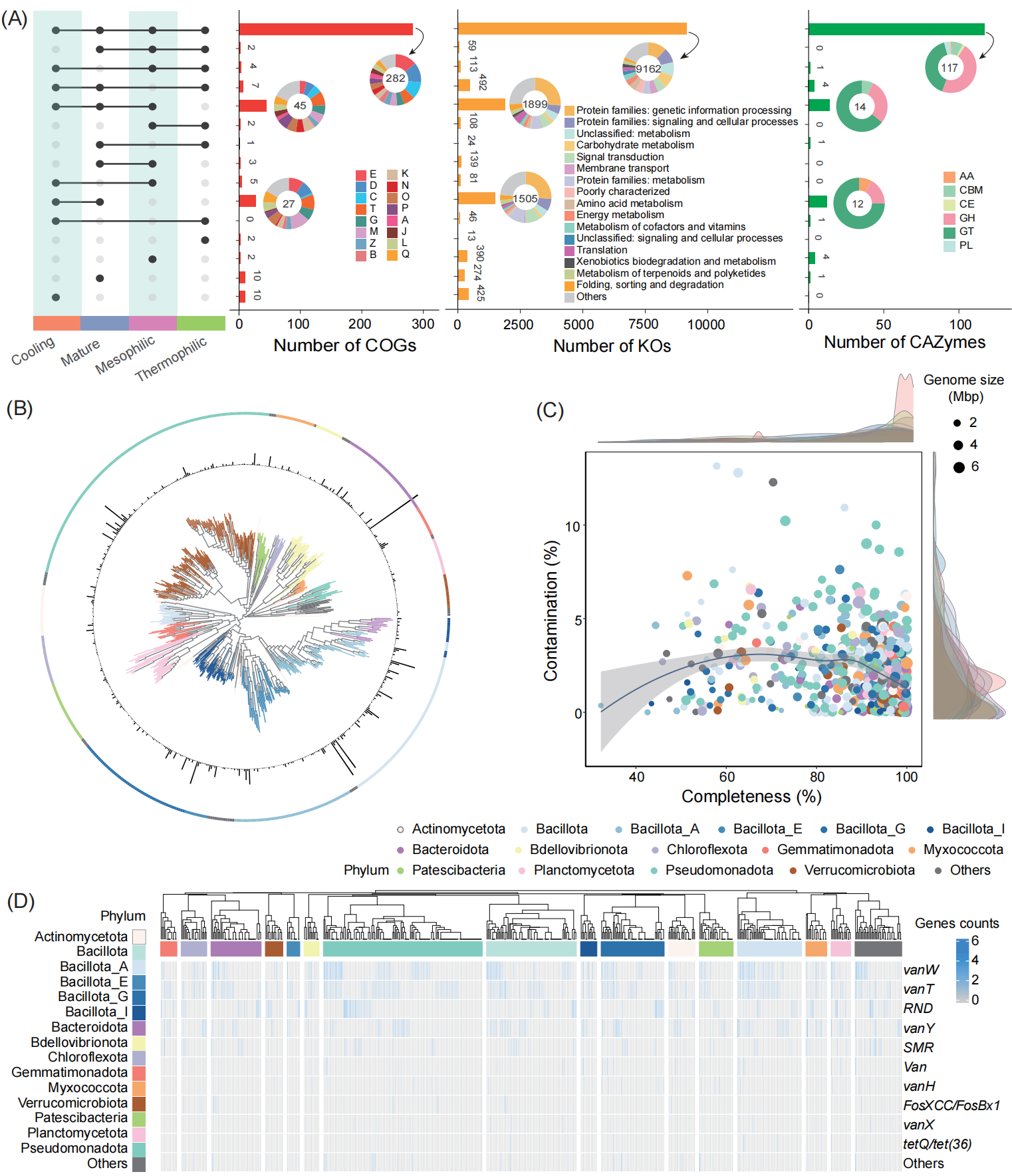


#### Figure S2. Functional annotation of assembled contigs and comparative analysis of MAGs in environmental microbiome samples. (A) Comparison of the levels of functional modules (COGs, KOs, and CAZy) of the microbiome across different groups. The left panel shows sets included in the intersection and independent sites, and the right bar or pie charts show the categories of the functional modules in these sets. The major enriched categories are shown in the legend. (B) Phylogenetic analysis and distribution of metagenome assembled genomes (MAGs) across different groups, highlighting evolutionary relationships of representative MAGs. The bar shows mean coverage of MAGs in all samples calculated by CoverM. (C) The distribution of completeness and contamination in MAGs, with the color of the points representing the phylum. (D) antibiotics resistance genes annotations within MAGs. The raw data from the Bioproject (PRJNA918803 and PRJNA917055) of NCBI with accession number in Table S2.

####
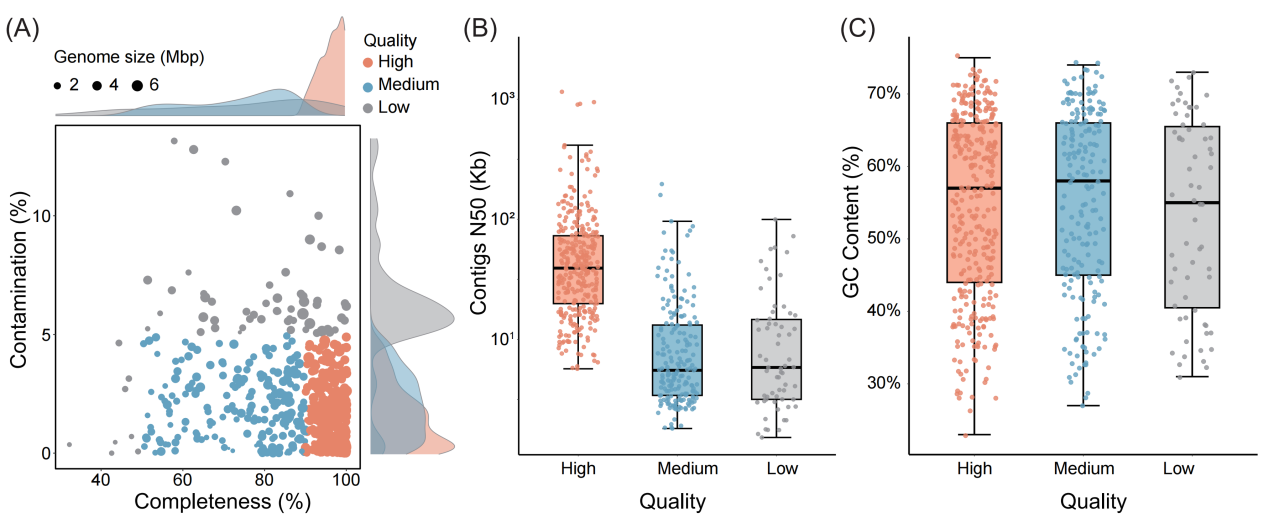


#### Figure S3. Genome quality of MAGs in environmental microbiome samples. (A) Completeness and contamination scores for all MAGs, colored by their quality classification category. (B) Contigs N50 distribution for all MAGs. (C) GC content for all MAGs. High: ≥ 90% completeness, ≤ 5% contamination. Medium: > 50% completeness, ≤ 5% contamination. Low: < 50% completeness.
